# MCT4 promotes cell proliferation and invasion of castration-resistant prostate cancer PC-3 cell line

**DOI:** 10.17179/excli2018-1879

**Published:** 2019-03-21

**Authors:** Qing Sun, Liang-liang Hu, Qiang Fu

**Affiliations:** 1 Department of Urology, Shandong Provincial Hospital Affiliated to Shandong University, Jinan, China; 2 Department of Urology, The Affiliated Huaian No.1 People’s Hospital of Nanjing Medical University, Huaian, China; 3 Department of Urology, Zaozhuang municipal Hospital, Zaozhuang, China

**Keywords:** prostate cancer, MCT4, aerobic glycolysis, siRNA, cell viability

## Abstract

Prostate cancer (PCa) is one of the leading causes of cancer-related death in men. Blocking androgen receptor (AR) signaling is an effective treatment strategy for the treatment of advanced metastatic disease of PCa in men. However, the method of blocking AR signaling is not suitable for castration-resistant prostate cancer (CRPC), and the treatment of CRPC is still clinically difficult. It has recently been reported that MCT4 is a plasma membrane transporter that mediates the secretion of lactic acid from aerobic glycolysis by cancer cells. Its expression is up-regulated in PCa and plays an important role in the carcinogenesis of PCa, but the underlying mechanism is hardly known. The MCT4 gene of PC-3 cell line was knocked down by siRNA, then MCT4 mRNA and protein was detected by real-time PCR and western blotting, respectively. CCK-8, Transwell migration assay, Flow cytometry, and TUNEL methods were used to detect the proliferation, invasion and apoptosis of PC-3 cells by MCT4 knockdown, and the expression of invasion-related proteins (MCT4) was detected by western blot analysis. The treatment of PC-3 with candidate MCT4 siRNAs led to marked inhibition of MCT4 expression in both mRNA and protein level. MCT4 knockdown inhibits PC-3 cell proliferation and facilitates apoptosis. Furthermore, MCT4 promoted the invasion capabilities of PC-3 cells by regulating invasion-related genes, such as VEGF, CD147, MMP2 and MMP9. In conclusion, MCT4 promotes oncogenic process of PCa may, as least partially, by inhibiting cell apoptosis and accelerating cell proliferation as well as invasion abilities of PC-3 cells. VEGF, CD147, MMP2 and MMP9 are important downstream genes of MCT4 in facilitating cell invasion.

## Introduction

Prostate cancer (PCa) is a hormone-dependent, common adult malignancy that is one of the leading causes of cancer-related death in men. An effective measure to treat the disease is to block androgen receptor (AR) signaling. The clinical application of enzalutamide, a clinically effective compound that blocks the AR axis, can significantly prolong the survival time of patients with advanced metastatic PCa (Bregni et al., 2018[2]). However, AR signaling-directed strategies are not curative in castration-resistant prostate cancer (CRPC) therapy (Bregni et al., 2018[2]; Prekovic et al., 2018[19]; Sharp et al., 2019[22]). Therefore, new strategies targeting alternative, fundamental cancer properties are urgently needed. 

Glucose metabolism present in cancerous tissues, manifested by an increase in aerobic glycolysis accompanied by increased production/secretion of lactic acid, is a key factor in the development of various cancers and involves therapeutic development (Hui et al., 2017[16]; Upadhyay et al., 2013[24]). Growing evidences have indicated that elevated glycolysis as well as excessive lactic acid secretion is common and clinically relevant in PCa (Christofk et al., 2008[6]; Cui et al., 2014[7]). Excessive lactic acid secretion facilitates tissue invasion, metastasis and neoangiogenesis, which can accelerate cancer progression (Augoff et al., 2015[1]; Riaz Rajoka et al., 2017[20]). 

The monocarboxylate transporters (MCTs) are a family of proton-linked plasma membrane transporters, and 14 members of the monocarboxylate transporter family (MCTs) have been identified in mammals, that carry molecules with monocarboxylates, such as lactate and pyruvate, across biological membranes. To date, only four (MCT1-MCT4) have been demonstrated to catalyze the proton-linked transport of monocarboxylates across the plasma membrane. MCTs 1-4 have distinct properties, tissue distribution and subcellular location, and are suitable for different metabolic effects (Fisel et al., 2018[11]; Halestrap, 2013[12]). MCT4, encoded by the SLC16A3 gene in human, predominantly mediates the cellular efflux of lactic acid/H+ (Dimmer et al., 2000[8]). So a new method for the treating CRPC may be obtained by targeting the MCT4 gene.

MCT4 is upregulated in tumor and has been shown to be related with poor prognosis in patients with multiple types of cancer (Chen et al., 2018[3]; Choi et al., 2018[5]; Fisel et al., 2013[10]; Todenhofer et al., 2018[23]), including PCa (Choi et al., 2016[4]; Pertega-Gomes et al., 2011[18]). In addition, upregulated MCT4 expression may be an important factor in cancer-stroma interactions facilitating PCa progression (Sanita et al., 2014[21]). This information suggests that the inhibition of the expression or function of MCT4 would provide a promising therapeutic strategy for a wide variety of neoplasms. 

In this study, we knockdown the expression of MCT4 using MCT4-targeting siRNAs in PC-3 cells, and found that MCT4 knockdown inhibits PC-3 cell proliferation and facilitate cell apoptosis. We also found that MCT4 involved the invasion abilities of PC-3 cells by regulating invasion-related genes, such as VEGF, CD147 and MMP9, which may explain, as least partially, how MCT4 promotes oncogenic process.

## Materials and Methods

### Cell cultures 

Human PC-3 CRPC cells were purchased from iCell Bioscience Inc. (Shanghai, China) and were routinely cultured in DMEM high glucose medium (Gibco) containing 10 % fetal bovine serum (FBS, Gibco) (containing 1.5 mM L-Glutamine, 100 U/ml penicillin, 100 μg/ml Streptomycin) at 37 °C with 5 % CO_2_ in a saturated humidity incubator.

### SiRNA design and transfection

Interfering RNA oligonucleotide sequences that specifically targeting MCT4 gene were designed and synthetized by Genepharma (Shanghai, China). The sequences of the synthetic oligonucleotides were as follows: (1) hMCT4-172: 5'-CAACCCUCCUGGCCAUGGGATT-3' and 5'-UCCCAUGGCCAGGAGGGUUGTT-3'; (2) hMCT4-1041: 5'-CCUACUCCGUCUACCUCUUTT-3' and 5'-AAGAGGUAGACGGAGUAGGTT-3'; (3) hMCT4-1377: 5'-GGCAACUUCUUCUGCAUUATT-3' and 5'-UAAUGCAGAAGAAGUUGCCTT-3'. Transfection of siRNAs was performed with Lipofectamine 2000 transfection reagents (Invitrogen) as recommended.

### Quantitative real-time PCR

Total RNA of PC-3 cells transfected with different RNA oligonucleotides was extracted using Ezol reagent (Genepharma), according to the manufacturer's instruction. The cDNA synthesis and real-time PCR were performed using Real-time Fluorescence Quantitative General Reagents (Genepharma), following manufacturer's instructions. The primers used for PCR were MCT4 (forward) 5'-GATGCGACCCACGTCTACAT-3', and (reverse) 5'-GTTGCCCAGCAGCAAAATCA-3', as well as GAPDH (forward) 5'-TCTCTGCTCCTCCTGTTCGA-3' and (reverse) 5'-GCGCCCAATACGACCAATC-3'. MCT4 mRNA expression levels were calculated by relative quantification (2^-ΔΔCt^) method. Experiments were performed in triplicate.

### Western blotting

Total protein was extracted using M-PER Mammalian Protein Extraction Reagent (Pierce, Grand Island, NY, USA) from PC-3 cells transfected with different RNA oligonucleotides, and protein concentration was determined using a BCA Protein Assay Kit (Pierce, Grand Island, NY, USA). The target protein was isolated by electrophoresis on a 10-12 % sodium dodecyl sulfate (SDS) polyacrylamide gel and the isolated protein was transferred to a polyvinylidene fluoride (PVDF) membrane (Millipore, Billerica, MA, USA). The membrane was then incubated overnight in primary antibody dilution at 4 °C followed by incubating with horseradish peroxidase-conjugated secondary antibody (Cell Signaling Technology) for 1 hour at room temperature. Chemiluminescence detection was performed with SuperSignal West Pico Chemiluminent Substrates (PIERCE) by exposure to an X-ray film. After development and fixing, the brands were photographed by a gel imaging analysis system and analyzed by Gel-Pro Analyzer software. GAPDH was used as an endogenous control for equal loading.

### CCK8 assay

The cell viability of the PC-3 cells transfected with siRNA was determined using CCK8 method. PC-3 cells (3×10^3^ cells) were seeded in each well of the 96-well plate. After the cells were adhered, a mixture containing 3 μl of siRNA oligo and 0.5 μl of lipofectamin 2000 was added into the 96-well plate. After 5 h of incubation, the transfection solution was discarded and 100 μl of DMEM medium containing 10 % FBS was added. Before the transfection of siRNA (0 h) and 24, 48, 72 h after transfection of siRNA, cells were cultured in 100 μl of fresh medium with 5 μl CCK8 for 2 h in the dark, respectively. The cell viability was calculated by the absorbance value (optical density: OD) at 450 nm.

### Apoptosis assays

Cell apoptosis was detected by flow cytometry and TdT-mediated dUTP Nick-End Labeling (TUNEL), respectively. For flow cytometry, PC-3 cells plated in 6-well plates were transfected with 30 μl siRNA oligo using 5 μl lipofectamin 2000. After 48 h of transfection, cells were harvested and apoptosis was measured using the Annexin V/PI Apoptosis Detection Kit (Mbchem) and flow cytometry. For TUNEL assay, apoptosis was analyzed using TUNEL cell apoptosis in situ detection kit (KeyGEN, Nanjing, China) according to the manufacturer's instructions. 

### Transwell migration assay

The Transwell chamber basement membrane (8 μm pores; Coring, NY, USA) was coated with 60 μl Matrigel (50 mg/L, BD Biosciences) and incubated at 37 °C for 4 h. After 6 h of transfection with si-MCT4, a number of 1×10^3^ cells were seeded in the upper chamber of the Transwell, and 700 μl of DMEM medium containing 20 % FBS was added to the lower chamber, and cells were cultured at 37 °C for 48 h. The invaded cells fixed with pre-cooled absolute ethanol for 10 min at -20 °C followed by being stained with 0.1 % crystal violet for 30 min at 37 °C. The invaded cells were observed under an inverted microscope and photographed.

### Statistical analysis

All data results in this study were shown as the mean ± SD. Data analysis was performed using GraphPad Prism 5. The Student's *t* test was performed to compare the significance of the differences between the two groups. A *P*-value of < 0.05 was considered statistically significant, which was indicated by *. And **, *P* < 0.01; and ***, *P* < 0.001.

## Results

### MCT4-targeting siRNAs effectively silence the expression of MCT4

It has been reported that MCT4 is upregulated in PCa, and that elevated expression of MCT4 protein in PCa is associated with the development of CRPC (Choi et al., 2016[4], 2018[5]). In this study, human CRPC PC-3 cell line was used as these cells present a distinct glycolytic metabolic profile, a property associated with MCT4 expression (Vaz et al., 2012[25]). 

**Figure 1:**
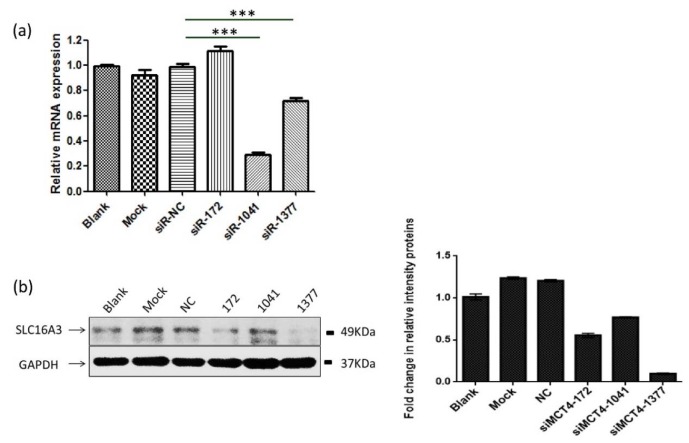
MCT4-targeting siRNAs effectively silence the expression of MCT4. (a) After 48 h transfection of MCT4 siRNAs into PC-3 cells resulted in reduced expression of MCT4 in mRNA levels. (b, c) Western blot analysis of the expression of MCT4 in PC-3 cells transfected with control siRNA and special MCT4 targeting siRNAs. The results were represented as the mean ± SD. ***, *P* < 0.001.

To investigate the effect of MCT4 silencing on PC-3 prostate cancer cells, the cells were transfected with MCT4-siRNA. Three MCT4-siRNAs give different efficiency knock down of gene expression. siRNA-1041 showed a high 65 % knockdown efficiency compared to only 25 % to siRNA-1377 in mRNA level. However, siRNA-1377 give a high 85 % efficiently knock compared to about 20 % to siRNA-1041 in protein level (Figure 1(Fig. 1)).

### Knockdown of MCT4 inhibits PC-3 cell proliferation but facilitate apoptosis

**Figure 2:**
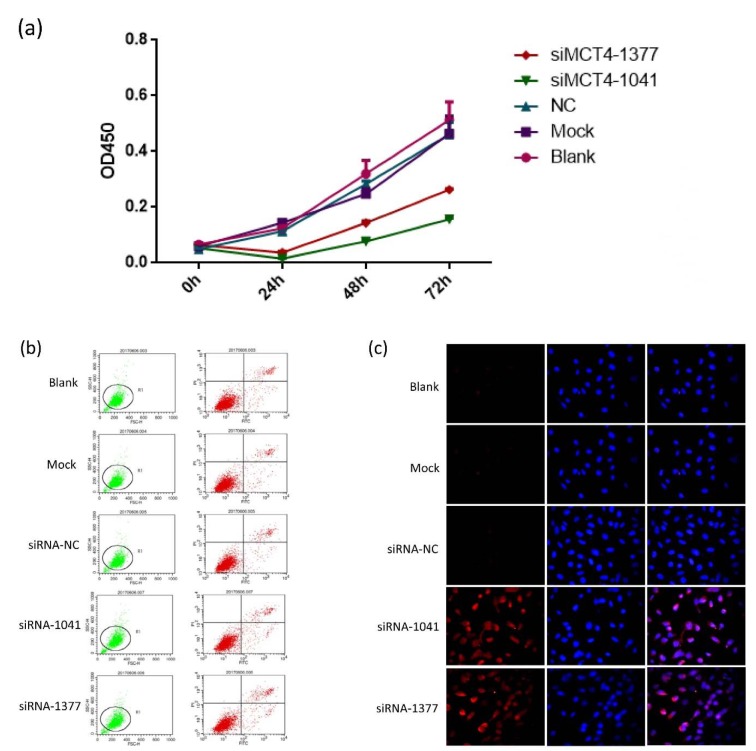
Knockdown of MCT4 inhibits PC-3 cell proliferation but facilitates apoptosis. (a) Cell proliferation was decreased in PC-3 cells transfected with candidate siRNAs, which persisted up to 72 h post transfection. Apoptosis in PC-3 cells transfected with candidate siRNAs was detected by Annexin V-FITC/PI double-labeled flow cytometry (b) and TUNEL assay (c).

After establishing siRNA efficacy, we assessed the phenotype changes induced by MCT4 knockdown in PC-3 cells. MCT4-siRNAs were transfected into PC-3 cells and then the proliferation of PC-3 cells were analyzed at 0 h, 24 h, 48 h and 72 h post-knockdown. We observed significant decreasing cell viability in MCT4 knockdown cells compared with scrambled controls (Figure 2a(Fig. 2)). 

The apoptosis of MCT4-silenced cells and scrambled control siRNA treated cells was examined by the percentage of propidium iodide, annexin-V-stained cells and TUNEL assay.

MCT4 siRNA treatment in PC-3 cells induced cell apoptosis (Figure 2b, 2c(Fig. 2)). These results showed that MCT4 promotes cell proliferation while inhibits PC-3 cells apoptosis.

### MCT4 facilitates invasion of PC-3 cells by regulating some molecules associated with VEGF/CD147/MMP9 signaling pathway

**Figure 3:**
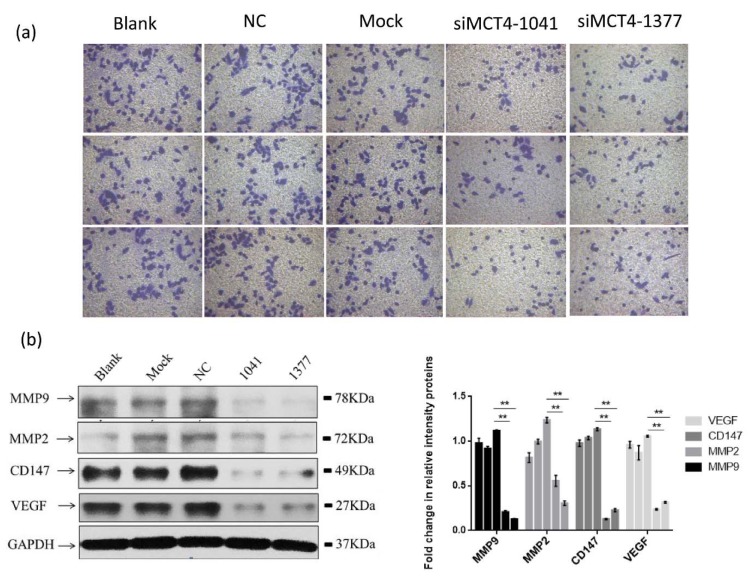
MCT4 facilitates invasion of PC-3 cells. (a) MCT4 knockdown inhibited PC-3 cells invasion as measured by Transwell assay. PC-3 cells were grown and transfected with negative control siRNA (siR-NC) or MCT4 siRNAs for 48 h. The graph summarizes the data from three independent experiments (right panel). *P < 0.05, **P < 0.01, ***P < 0.001. (b) MCT4 knockdown regulated expression of some proteins associated with cell invasion, especially VEGF, CD147, MMP2 and MMP9.

Here, we evaluated the effects of MCT4-siRNA in PC-3 cell lines, focusing on invasion. MCT4 siRNA transfected PC-3 cells showed a lower invasive ability compared with control cells through a matrigel invasion assay (Figure 3a(Fig. 3), *P* < 0.01).

Considering that MCT4 promotes invasion of cancer cells, we deemed it prudent to examine the expression of invasion-related genes in MCT4 knockdown cells. We conducted western blot analysis using antibodies specific for these proteins to confirm that MCT4 facilitates invasion of PC-3 cells. As shown in Figure 3b(Fig. 3), PC-3 cell invasion-related genes VEGF, CD147, MMP2 and MMP9 in MCT4 siRNA transfected PC-3 cells showed a low expression level compared to the control cells.

## Discussion

Compared with normal tissues, the energy metabolism of cancer tissues is special, with the characteristics of up-regulation of glycolysis and excessive secretion of lactic acid (Hanahan and Weinberg, 2011[14]). Due to the accumulation of lactic acid in the tumor, the extracellular fluid of the tumor is acidic, which accelerates invasion and metastasis of the tumor. Besides, an acidic tumor microenvironment increases the resistance of the cancer cells to chemotherapeutic drugs (Augoff et al., 2015[1]; Levine and Puzio-Kuter 2010[17]; Riaz Rajoka et al., 2017[20]). MCT4 can function as a lactate exporter or importer, and lactate could accumulate while MCT4 inhibition (Todenhofer et al., 2018[23]). The increase in MCT4 expression is associated with clinical manifestations and may play an important role in the progression of the disease to the late stage or the enhancement of cancer cell invasion (Choi et al., 2016[4], 2018[5]; Hao et al., 2010[15]; Pertega-Gomes et al., 2011[18]). 

To investigate the function of MCT4 in PCa, we silence MCT4 expression using three MCT4 targeting short interfering RNAs (siRNAs) in PC-3 cells. MCT4 inhibition appears to inhibit castration-resistant prostate cancer PC-3 cell line proliferation, leading to cell apoptosis (Figure 2(Fig. 2)). Furthermore, *in vitro* invasion of MCT4 knockdown PC-3 cells decreased, indicating that lactate secretion may be involved in local tissue cell invasion (Doherty and Cleveland 2013[9]; Han et al., 2013[13]). MCT4 inhibition appears to inhibit castration-resistant prostate cancer PC-3 cell line invasion and migration, leading to a low transcription and expression levels of invasion-related genes in castration-resistant prostate cancer (Figure 3(Fig. 3)). However, the precise mechanism of the inhibition of down-regulation of MCT4 on proliferation, invasion of PC-3 cells, and other aspects of cancer biology, such as tumorigenesis, need to be elucidated further. In particular, a proteomics or metabolomics method could be used as a tracer to determine what plays the crucial role in PCa carcinogenesis. 

Abnormal glucose metabolism has also been reported as clinically relevant and functionally significant in many types of cancer. Glycolytic metabolic mediator plays an important role in the cancer cell metabolism, proliferation and invasion, could be a new potential target for anti-castration-resistant prostate cancer therapy. In addition, treatment efficacy could also be increased using a combination strategy with other therapeutics currently used for treating CRPC (such as second-generation hormone therapies or docetaxel). Alternatively, combination with other modulators of glucose metabolism (such as metformin or mitochondrial inhibitors) might result in a more complete blockade of glucose utilization by cancer cells.

## Acknowledgements

This study was supported by the grants from Natural Science Foundation of Shandong Province (Grant No.: ZR2017BH036) and Shandong Medical Science and Technology Development Program (Grant No.: 2017WS289).

## Conflict of interest

The authors declare that they have no conflict of interests.
